# Enhancing the Output Performance of Fiber-TENG Through Graphite Doping and Its Application in Human Motion Sensing

**DOI:** 10.3390/s25206409

**Published:** 2025-10-17

**Authors:** You-Jun Huang, Jen-I Chuang, Chen-Kuei Chung

**Affiliations:** Department of Mechanical Engineering, National Cheng Kung University, Tainan 701, Taiwan

**Keywords:** energy harvester, graphite, fiber, triboelectric nanogenerators, force sensors

## Abstract

Triboelectric nanogenerators (TENG) are mechanical energy harvesters characterized by high sensitivity and simple structure and are currently being widely developed for use in human body motion sensing. Among them, fiber-based TENGs (FTENG) are particularly suitable for wearable human motion sensors due to their unique structure, which offers flexibility, high durability, and comfort. However, studies involving doping to further modify the electrical output characteristics of FTENGs are very limited. Here, we propose an innovative approach that combines graphite (GP) doping with fiber-based TENG fabrication, successfully developing a graphite-doped polyester fiber-based TENG (GP@PET-TENG). Proper graphite doping can increase the amount of transferred charge and thus improve the output electrical performance of TENG, but this method has rarely been explored in FTENG. With the incorporation of 3%wt graphite, the open-circuit voltage of the GP@PET-TENG increased from 103.3 V to 202.1 V, and the short-circuit current increased from 60.7 μA to 105.1 μA, compared to the pure polyester fiber based TENG (PET-TENG). The device achieved a maximum output power of 4.15 mW (2.59 W/m^2^), demonstrates the capability to charge various capacitors, and successfully lit up 200 LEDs. By attaching the GP@PET tribo-layer to human skin, a single-electrode mode TENG can be formed, which captures the subject’s motion signals through skin contact and separation, converting them into voltage outputs. In fist-clenching and wrist-bending tests, motion-induced voltage signals up to 0.6 V were recorded, demonstrating the potential applications in rehabilitation assistance and mechanical control.

## 1. Introduction

With recent advancements in semiconductor technology reducing critical dimensions and costs, wearable sensors have become smaller and more widespread. This has enabled the real-time capture and recording of previously hard-to-measure human motion signals and data, such as respiratory rate and blood oxygen levels. Such data can be used for health monitoring, exercise tracking, rehabilitation, medical applications, and even mechanical control [1,2,3]. For example, human body motion sensors record the distribution of force exerted by human steps for rehabilitation purposes or transmit motion data to a computer for data control in real-time [4,5]. Traditional human body motion sensors often use capacitive and resistive devices that require an external energy supply [6,7]. As the precision of human motion measurement increases, more sensors are required in the system, leading to higher energy consumption. Besides the energy issue, current power storage devices cannot sustain such sensor systems. Therefore, finding an alternative to traditional mechanical sensing devices is crucial for developing wearable sensors [8,9,10]. Mechanical energy harvesters, which convert mechanical energy into electrical signals and power, such as electro-mechanical generators and piezoelectric nanogenerators, are considered future substitutes for sensors [11]. TENG, known for its high mechanical sensitivity, material flexibility, and lightweight properties, is particularly viewed as an essential technology to replace traditional wearable sensors [12]. TENG operates through four mechanisms [13], with vertical contact-separation [14,15] and single-electrode modes [16] being widely used as mechanical sensors due to their strong correlation between output voltage and applied force, and high linearity [17,18]. FTENGs are a type of TENG that incorporate fiber structures or fiber-based into polymer-based materials to enhance both the mechanical robustness and electrical output performance of the device. FTENGs are commonly fabricated by either electrospinning polymers such as polyvinylidene fluoride (PVDF) into fibers [19] for textile formation or embedding commercial fiber fabrics such as cotton into polymer materials [20]. Owing to their lightweight nature, high durability, stretchability, and flexibility, they are considered one of the most promising TENG types for the development of wearable sensors [21,22,23] and have been applied in human motion sensing [24,25]. Fabricating tribo-layers of FTENGs by coating polymer materials onto fiber provides benefits of rapid, low-cost processing [26,27,28]. However, further enhancement of their electrical output remains challenging. Material doping presents an alternative strategy by modifying the electrical properties of the tribo-layers [29,30,31,32]. For instance, conductive material doping has been reported to reduce charge transfer resistance and enhance both transferred and induced charge density [33,34]. Compared with other conductive dopants such as CNTs [35] and metallic materials [36], micron-sized graphite particles offer advantages of low cost, easy availability, and low environmental impact; yet this approach has rarely been explored in FTENGs.

In this study, we propose an innovative method to enhance FTENG performance by doping PDMS with varying concentrations of micron-sized graphite and coating it onto polyester fabric to fabricate GP@PET-TENGs. GP@PET-TENG doped with 3%wt graphite exhibited the highest output performance, with an open-circuit voltage of 202.1 V and a short-circuit current of 105.1 μA which representing 1.96 times and 1.73 times increase, respectively, compared to the pure PET-TENG (103.3 V and 60.7 μA). GP@PET-TENG achieved a maximum output power of 4.15 mW (2.59 W/m^2^) at a load resistance of 1 MΩ and which is enable to charge various capacities from 0.33 μF to 2.2 μF over 1.0 V and light up to 200 LEDs at once. By attaching the GP@PET tribo-layer to human skin, it forms a single-electrode mode TENG capable of effectively capturing motion signals generated from bending at the third finger joint and wrist. These mechanical movements are converted into electrical signals with a maximum output voltage of 0.6 V. In the future, with the integration of signal processing circuits and electronic control modules, these signals can be further converted into meaningful human motion data and control signals for practical applications.

## 2. Experimental Procedures

### 2.1. Fabrication of GP@PET-TENG

**Figure 1:**
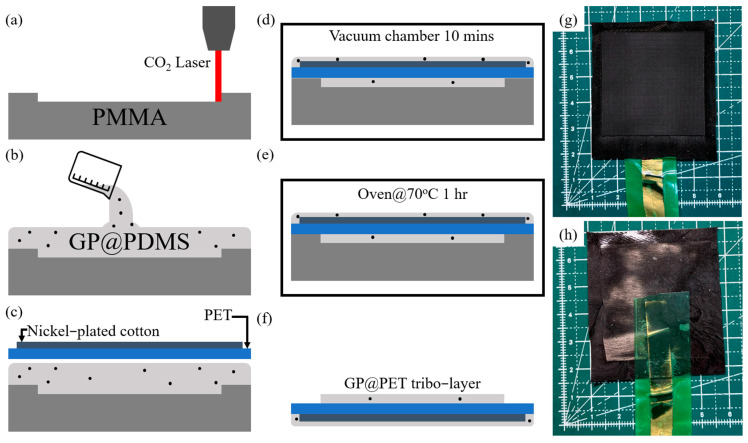
Process flow of GP@PET-TENG fabrication: (**a**) Use a CO_2_ laser to cut a 40 × 40 × 1 mm^3^ groove on an 50 × 50 × 5 mm^3^ PMMA mold; (**b**) Pour a mixture of PDMS and 1~5%wt graphite powder into the PMMA mold; (**c**) Place the PET, pre-attached with nickel-coated fabric, onto the GP@PDMS and allow it to sink into the mixture; (**d**) Place the sample and mold into a vacuum chamber for 10 min to remove air bubbles; (**e**) Place the sample and mold into an oven at 70 °C for 1 h to cure; (**f**) Demold to obtain the GP@PET tribo-layer; Photograph of: (**g**) the front side and (**h**) the back side of the fabricated GP@PET tribo-layer.

GP@PET-TENG is composed of an aluminum electrode and graphite-doped PDMS coated PET fibers. The aluminum electrode is commercially available low-purity aluminum, ready for use upon purchase. This section focuses on the fabrication process of the graphite-doped PDMS-coated PET fibers. To create a sufficient PDMS thickness, a PMMA mold with a 50 × 50 × 5 mm^3^ dimension was used. A 1 mm deep groove was made in the PMMA using a CO_2_ laser, creating a 40 × 40 mm^2^ recess, as shown in Figure 1a. Next, 1~5%wt micron-sized graphite particles were added to the PDMS fluid mixed with a curing agent. This fluid was then poured into the PMMA mold, as depicted in Figure 1b. The 50 × 50 mm^2^ PET, pre-attached to a 40 × 40 mm^2^ nickel-coated cotton cloth electrode, was submerged into the PDMS fluid, allowing it to infiltrate and coat the PET fiber structure, as shown in Figure 1c. The sample and mold were placed in a vacuum chamber for 10 min to remove air bubbles from the PDMS and PET, as shown in Figure 1d. The sample and mold were then placed in an oven at 70 °C for 1 h to cure the PDMS, as shown in Figure 1e. Finally, the sample was demolded, and excess PDMS was trimmed to obtain the graphite-doped Polyester fiber based tribo-layers (GP@PET tribo-layers), as shown in Figure 1f. Figure 1g,h present the front and back sides of photographs of the fabricated GP@PET tribo-layers, respectively. The yellow reflective strip underneath corresponds to the copper tape, which functions as the lead for the nickel-coated fabric electrode in subsequent electrical connections. The green tape is applied to mechanically protect the copper tape during operation. By replacing the curing agent with pure PDMS in the same fabrication process, polyester fiber-based tribo-layers (PET tribo-layers) without graphite doping can be obtained.

### 2.2. The Experiment and Measurement of Various PET-TENG

**Figure 2:**
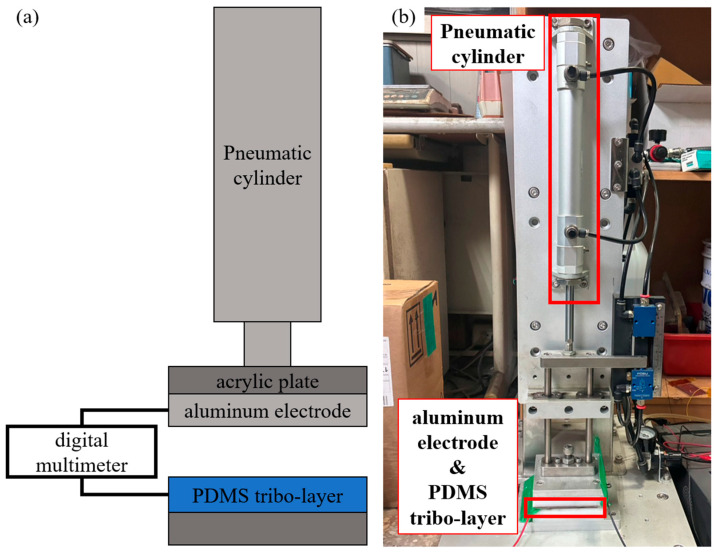
Platform used for evaluating the output performance of TENG: (**a**) Schematic diagram of the setup and (**b**) corresponding photograph. The platform is driven by a pneumatic cylinder operating at a frequency of about 7 Hz and a force of 50 N. The PDMS tribo-layer of the TENG is fixed on the stationary base, while the aluminum electrode and acrylic plate are mounted on the moving part to form a vertical contact–separation mode TENG with a stroke of 5 mm. The two electrodes are connected to a digital multimeter (DMM6500) to measure the open-circuit voltage and short-circuit current.

To evaluate and compare the output performance of various PET-TENGs, a digital multimeter (DMM6500 6.5 Digit Multimeter, Keithley, Solon, OH, USA) was used to measure the electrical output signals, while a pneumatic actuation platform provided a stable and repeatable external force. On the platform, the PDMS tribo-layer of the TENG was fixed onto the stationary base of the pneumatic cylinder (RHCL25-100, SMC, Chiyoda, Tokyo, Japan) using an acrylic plate, and the aluminum electrode, serving as the other tribo-layer, was mounted on the moving shaft. The measurements were conducted under a stroke length of 5 mm, with a periodic force of 50 N at a frequency of 7 Hz, as shown in Figure 2a,b. The measurements in this experiment were all conducted under an environment with a temperature of 25 °C and a humidity of 62%.

## 3. Results and Discussion

**Figure 3:**
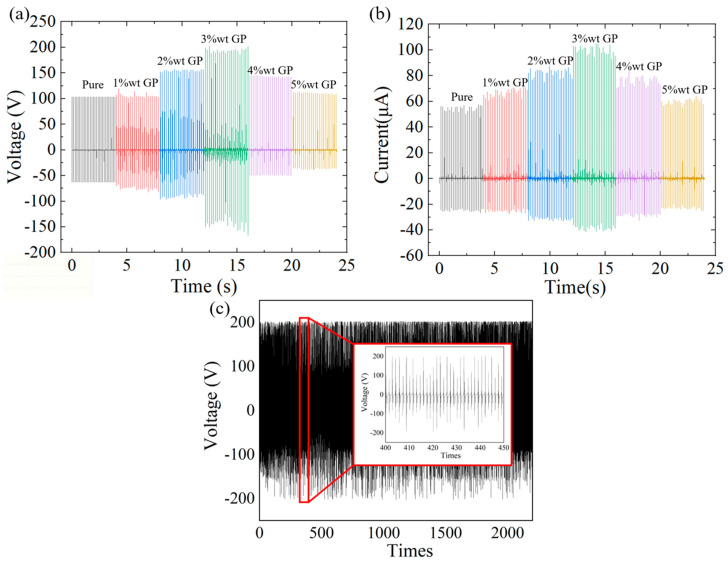
Comparison of output (**a**) Voc and (**b**) Isc of PET-TENGs with different graphite doping concentrations, and (**c**) the output voltage of 3%wt GP@PET-TENG under over 2000 cycles of contact–separation. The Voc values for PET-TENGs with 0% (pure), 1%, 2%, 3%, 4%, and 5% wt graphite doping are 103.3 V, 118.8 V, 157.4 V, 202.1 V, 145.5 V, and 113.0 V, respectively; the corresponding Isc values are 60.7 μA, 71.1 μA, 87.2 μA, 105.1 μA, 83.7 μA, and 64.3 μA. Among them, the 3% wt GP@PET-TENG exhibits the highest output in both Voc and Isc, representing a 1.96-fold and 1.73-fold increase, respectively, compared to the undoped PET-TENG. In (**c**), the output voltage of the 3% wt GP@PET-TENG remains stable over 2000 cycles of contact–separation, demonstrating its durability under repeated loading.

In TENG research, the open-circuit voltage (Voc) and short-circuit current (Isc) are commonly used as key indicators to evaluate output performance. To investigate the effect of different graphite doping concentrations on PET-TENGs, we first measured the Voc and Isc of the undoped PET-TENG (pure) as well as GP@PET-TENGs doped with various graphite weight percentages (1%wt, 2%wt, 3%wt, 4%wt, and 5%wt). The measured Voc values were 103.3 V, 118.8 V, 157.4 V, 202.1 V, 145.5 V, and 113.0 V, respectively, while the corresponding Isc values were 60.7 μA, 71.1 μA, 87.2 μA, 105.1 μA, 83.7 μA, and 64.3 μA, as shown in Figure 3a,b. These results indicate that the PET-TENG with 3%wt graphite doping exhibited the highest output performance in both Voc and Isc, representing improvements of 196% and 173%, respectively, compared to the undoped PET-TENG. Additionally, by testing the Voc of the 3%wt GP@PET-TENG over more than 2000 cycles of contact–separation, it was observed that the output did not show any significant degradation. This demonstrates that the 3%wt GP@PET-TENG has a durability exceeding 2000 cycles, and during this period, neither the PDMS film nor the graphite particles suffered damage, leakage, or failure, as shown in Figure 3c.

Given that the composite thin-film TENG structure belongs to the conductor–dielectric contact mode [37], this phenomenon can be explained using the V–Q–X relationship [37], which describes the relationship among the output voltage (V), transferred charge (Q), and the separation distance (X) between the two tribo-layers as follows:

(1) $$V=\frac{-Q}{A\varepsilon_{0}}\left(\frac{d}{\varepsilon_{r}}+x\left(t\right)\right)+\frac{\sigma \cdot x\left(t\right)}{\varepsilon_{0}}$$

where ε_0_ represents the vacuum dielectric constant, A is the surface area, ε_r_ is the relative dielectric constant of the composite material, d is the thickness of the dielectric layer, and σ is the surface charge density. In the open circuit condition, there is no charge transfer, that is, Q is 0, and the Voc is given by:

(2) $$V_{oc}=\frac{\sigma \cdot x\left(t\right)}{\varepsilon_{0}}$$

The V_OC_ is proportional to the triboelectric charge density (σ). Having a higher triboelectric charge density will increase the output voltage.

Under short-circuit conditions, there is no output voltage and the I_SC_ is given by:

(3) $$I_{sc}=\frac{d}{dt}Q_{sc}=\frac{d}{dt}\left[\frac{A\sigma \cdot x\left(t\right)}{\frac{d}{\varepsilon_{r}}+x\left(t\right)}\right]=\frac{A\sigma \left(\frac{d}{\varepsilon_{r}}\right)}{{\left(\frac{d}{\varepsilon_{r}}+x\left(t\right)\right)}^{2}}\;\frac{dx}{dt}$$

In addition, the triboelectric charge density σ is proportional to the dielectric constant (εr),

(4) $$\sigma =\frac{\varepsilon_{0}\varepsilon_{r}V}{d}$$

Since the thickness (d), distance (x(t)), and contact area (A) are consistent across different PET-TENG samples, the variations in voltage and current output can be attributed to differences in the charge density (σ) of the tribo-layers. In dielectric tribo-layers, doping with a moderate amount of conductive material can enhance the material’s conductivity, thereby improving the efficiency of charge transfer. However, excessive doping may reduce the dielectric properties of the tribo-layer, weakening its ability to retain electrostatic potential. Therefore, appropriate doping contributes to an increase in both the charge density (σ) and the relative permittivity (εr), ultimately enhancing the output voltage and current of the TENG. In this study, micron-sized graphite particles were incrementally doped into the PDMS film to enhance charge transport efficiency and thereby improve the electrical output performance of the TENG. However, when the graphite doping concentration exceeded 3 wt%, the dielectric properties of the PDMS film were adversely affected, leading to a decline in output performance. A doping ratio of 3 wt% was identified as the optimal threshold, resulting in the best performance in the GP@PET-TENG. This is because when graphite, a conductive material, is doped into the dielectric material PDMS, the graphite reduces the impedance to charge flow within the GP@PDMS tribo-layer for increasing the amount of charge generated during the contact separation process, ultimately reflecting an increase in the open-circuit voltage and short-circuit current. Therefore, doping pure PDMS with graphite leads to improved output electrical performance. However, as the graphite doping concentration increases, exceeding 3 wt%, the relative permittivity of the GP@PDMS tribo-layer decreases significantly. This also reduces the amount of charge that can be generated during contact separation under the same conditions, further reducing the open-circuit voltage and short-circuit current. Increasing the graphite doping concentration at any concentration reduces both the impedance to charge flow within the GP@PDMS tribo-layer and the relative permittivity, but the effects of these two factors vary at different concentrations.

**Figure 4:**
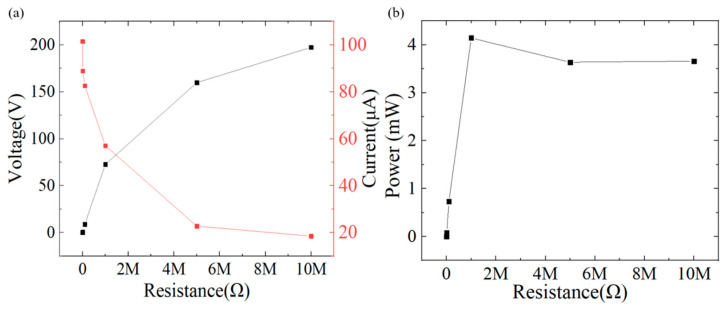
(**a**) Output voltage and current trends of the 3%wt GP@PET-TENG under different external loads. At an external load of 1 MΩ, the 3%wt GP@PET-TENG achieves its maximum output power of 4.15 mW (2.59 W/m^2^), as calculated using the formula P = I × V; (**b**) The corresponding output powers under different load resistances are 228.38 nW (142.75 μW/m^2^) @ 1 kΩ, 769 μW (480.63 mW/m^2^) @ 10 kΩ, 728.78 μW (455.49 mW/m^2^) @ 100 kΩ, 4.15 mW (2.59 W/m^2^) @ 1 MΩ, 3.64 mW (2.28 W/m^2^) @ 5 MΩ, and 3.66 mW (2.29 W/m^2^) @ 10 MΩ.

By measuring the output voltage and current of the TENG under different external load of resistances, the maximum output load and electrical performance can be determined with formula P = I × V. The 3%wt GP@PET-TENG achieved its highest output power of 4.15 mW (2.59 W/m^2^) at an external load of 1 MΩ with 72.66 V and 57.05 μA, as shown in Figure 4a. The output powers at different load resistances were measured as follows: 228.38 nW (142.75 μW/m^2^) at 1 kΩ, 769 μW (480.63 mW/m^2^) at 10 kΩ, 728.78 μW (455.49 mW/m^2^) at 100 kΩ, 3.64 mW (2.28 W/m^2^) at 5 MΩ, and 3.66 mW (2.29 W/m^2^) at 10 MΩ, as shown in Figure 4b.

**Figure 5:**
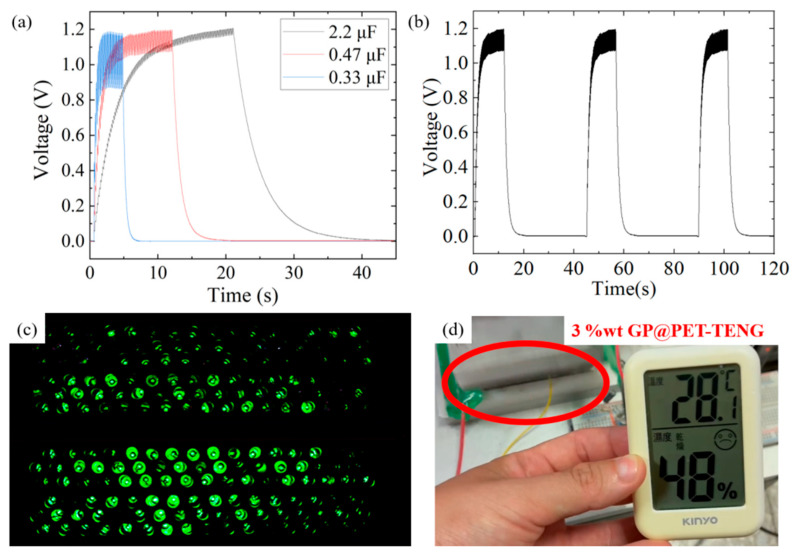
Performance test of the 3%wt GP@PET-TENG in driving electronic components: (**a**) The 3%wt GP@PET-TENG successfully charges capacitors of 2.2 μF, 0.47 μF, and 0.33 μF to 1.2 V, 1.1 V, and 1.0 V within 23 s, 5 s, and 1 s, respectively, and discharges them within 18 s, 5 s, and 1 s; (**b**) Repeated charge–discharge voltage cycles of the 0.47 μF capacitor demonstrate the high repeatability of the 3%wt GP@PET-TENG in charging and discharging capacitors; (**c**) The 3%wt GP@PET-TENG is capable of simultaneously lighting up 200 LEDs; (**d**) After charging a 0.47 μF capacitor, the stored energy is used to power an electronic temperature–humidity sensor, demonstrating the potential of the 3%wt GP@PET-TENG to drive small electronic sensing devices.

To further evaluate the electrical driving capability of the 3%wt GP@PET-TENG, various capacitors were connected in parallel with a 1 MΩ load resistor via a bridge rectifier and a circuit switch, and then charged/discharged by the 3%wt GP@PET-TENG. The device successfully charged 2.2 μF, 0.47 μF, and 0.33 μF capacitors to stable voltages of 1.2 V, 1.1 V, and 1.0 V within 23 s, 5 s, and 1 s, respectively, as shown in Figure 5a. Due to their higher charge storage capacity, larger capacitors tend to exhibit higher terminal voltages upon full charging. After switching off the circuit, the corresponding discharge times were 18 s, 5 s, and 1 s. This charge–discharge behavior is highly repeatable, as shown in Figure 5b. Additionally, the 3%wt GP@PET-TENG demonstrated the capability to instantaneously light up 200 LEDs with each tapping cycle, as shown in Figure 5c. These results confirm the excellent electrical output and component-driving capability of the 3%wt GP@PET-TENG. In addition, the 3 wt% GP@PET-TENG was connected across a 0.47 μF capacitor and linked to a miniature electronic temperature–humidity sensor. Once the capacitor was fully charged and the voltage stabilized, the 3 wt% GP@PET-TENG successfully powered the sensor, demonstrating its potential for driving small electronic sensing devices, as shown in Figure 5d.

**Figure 6:**
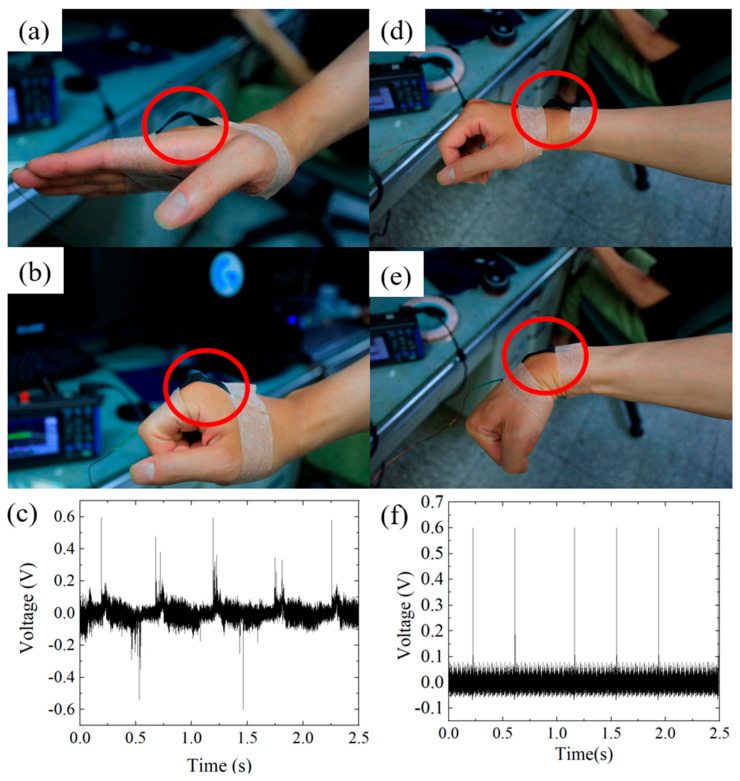
A single-electrode mode TENG is formed by combining the 3%wt GP@PET tribo-layer with human skin, and used to capture joint bending motions and convert them into voltage signals. (**a**) The 3%wt GP@PET tribo-layer is attached to the back of the subject’s third finger joint; (**b**) when the subject clenched their fist, the skin contacts the tribo-layer and separates upon relaxation, completing a contact-separation cycle; (**c**) a voltage signal with a peak output of 0.6 V is generated during the motion; (**d**) the tribo-layer is also attached to the back of the wrist joint; (**e**) when the wrist bends, contact and subsequent separation occur between the skin and the tribo-layer; (**f**) this motion similarly generates a voltage output of 0.6 V. These signals can be further utilized for human motion tracking and control applications in the future.

Finally, the 3%wt GP@PET tribo-layer was utilized as a human body motion sensor by attaching it to the skin to detect stretching and contraction during joint bending. Since human skin is a charge-releasing material in the tribo-pair, it can form a single-electrode mode TENG with the 3%wt GP@PET tribo-layer. The sensor was attached to the back of the third knuckle of the finger and the back of the wrist joint, as shown in Figure 6a,d. When the subject clenched their fist or bent their wrist, the tribo-layer made contact with the skin, as shown in Figure 6b,e; when the fist or wrist was straightened, the tribo-layer separated from the skin, completing a contact-separation cycle, as shown in Figure 6a,d. In each bending and straightening motion, the 3%wt GP@PET tribo-layer consistently captured the motion signals, generating electrical outputs of up to 0.6 V for both fist and wrist movements, as shown in Figure 6c,f. When the 3%wt GP@PET tribo-layer is attached to the third knuckle, fist clenching and relaxing cause not only contact-separation cycles with the skin but also induce lateral deformation of the tribo-layer. In comparison, the wrist joint exhibits more stable output signals during flexion and extension. With the integration of electronic control modules and signal processing circuits, these signals can be further utilized as effective inputs for motion monitoring and control applications.

## 4. Conclusions

To rapidly and cost-effectively enhance the output performance of flexible FTENGs, this study uniquely introduces graphite doping to further improve their electrical output. Among various graphite doping concentrations, the GP@PET-TENG with 3%wt graphite exhibited the highest performance, achieving an open-circuit voltage (Voc) of 202.1 V and a short-circuit current (Isc) of 105.1 μA. It also reached a maximum output power of 4.15 mW (2.59 W/m^2^) under a 1 MΩ external load. The 3%wt GP@PET-TENG demonstrated the ability to charge a 2.2 μF capacitor to 1.2 V within 23 s and discharge it within 18 s, and successfully powered 200 LEDs with a single tap. When the 3%wt GP@PET tribo-layer was attached to the back of a subject’s third finger joint and wrist, it formed a single-electrode mode TENG capable of capturing motion signals during finger and wrist bending. These signals were converted into output voltages up to 0.6 V. By integrating this TENG with signal processing circuits and control units, human motion signals can be effectively captured and utilized for motion control and real-time monitoring applications.

## Data Availability

Data are presented in the coauthors’ research results and schematic drawings available on request.
